# Brainstem abnormalities and vestibular nerve enhancement in acute Neuroborreliosis

**DOI:** 10.1186/1756-0500-6-551

**Published:** 2013-12-21

**Authors:** Nadja A Farshad-Amacker, Hans Scheffel, Thomas Frauenfelder, Hatem Alkadhi

**Affiliations:** 1 Diagnostic and Interventional Radiology Department, University Hospital of Zurich, Raemistrasse 100, Zurich 8091, Switzerland

**Keywords:** Borreliosis, Brain stem, MRI, Neuroborreliosis, Vestibular nerve enhancement

## Abstract

**Background:**

Borreliosis is a widely distributed disease. Neuroborreliosis may present with unspecific symptoms and signs and often remains difficult to diagnose in patients with central nervous system symptoms, particularly if the pathognomonic erythema chronica migrans does not develop or is missed. Thus, vigilance is mandatory in cases with atypical presentation of the disease and with potentially severe consequences if not recognized early. We present a patient with neuroborreliosis demonstrating brain stem and vestibular nerve abnormalities on magnetic resonance imaging.

**Case presentation:**

A 28-year-old Caucasian female presented with headaches, neck stiffness, weight loss, nausea, tremor, and gait disturbance. Magnetic resonance imaging showed T2-weighted hyperintense signal alterations in the pons and in the vestibular nerves as well as bilateral post-contrast enhancement of the vestibular nerves. Serologic testing of the cerebrospinal fluid revealed the diagnosis of neuroborreliosis.

**Conclusion:**

Patients infected with neuroborreliosis may present with unspecific neurologic symptoms and magnetic resonance imaging as a noninvasive imaging tool showing signal abnormalities in the brain stem and nerve root enhancement may help in establishing the diagnosis.

## Background

Borreliosis (Lyme disease) is a world wide distributed disease, transmitted by ticks, with particular focus in Europe and North America. Borreliosis can be classified in three clinical stages (Table 1). Only 40-60% of patients present with erythema chronicum migrans as an initial pathognomonic sign (stage I) [1]. Flu-like symptoms, such as fever, headaches, neck stiffness and arthralgia may be followed by meningeal irritation, encephalitis and/or neuritis (stage II) and further, cardiac involvement may develop. The final stage (stage III) may include arthritis, encephalomyelitis and acrodermatitis chronica atrophicans [2] (Table 1).

**Table 1:** Stages of borreliosis

**Stages**	**Time course**	**Clinic manifestation**
Stage I	Days- weeks	Erythema chronicum migrans
Stage II	Weeks to < 6 months	Flue-like symptoms (Fever, arthralgias, headaches, neck stiffness, musculoskeletal pain, fatigue)
		Early CNS- infection (meningoencephalitis, neuritis)
		Cardiac involvement (AV- block, peri- myocarditis)
Stage III	> 6 months	Arthritis
		Encephalopathy (affecting mood, memory and sleep)
		Acrodermatitis chronica atrophicans

The diagnosis of neuroborreliosis is often challenging, particularly if the pathognomonic erythema chronicum migrans does not develop or is missed. Thus, it seems emergent to call for vigilance in case of unspecific presentations of the disease which might have devastating consequences if not recognized and treated early. In neuroborreliosis, magnetic resonance imaging (MRI) may show meningeal and cranial nerve enhancement after contrast administration [3-5] and/or signal abnormalities in the white matter of the brain, although rarely reported [6].

We present a 28-year-old patient infected with neuroborreliosis in whom MRI showed T2-weighted (T2w) hyperintense signal alterations in the pons and vestibular nerves as well as bilateral post-contrast vestibular nerve root enhancement.

## Case presentation

A 28-year-old Caucasian female presented with headaches and fever of unknown origin lasting for two weeks. The patient noticed a tick bite two weeks prior to the onset of fever and headaches. Past history and family history were otherwise unremarkable.

At the time of an ambulatory exam by a general practitioner (about three weeks after the tick bite), laboratory investigation e.g. complete blood cell count (CBC), C-reactive protein (CRP), electrolytes, liver enzymes, albumin, creatinin, lactate dehydrogenase (LDH), thyroid stimulating hormone (TSH), free thyroxine (free T4), glucose and Borrelia serum screening test were shown within normal limits. No abnormalities of the skin were noted. The patient was treated with non-steroidal analgetics and returned home.

The fever resolved spontaneously after two weeks (four weeks after the tick bite), however, the headaches remained and were further accompanied by neck stiffness, fatigue, nausea, weight loss of 5 pounds with decreased appetite, an intentional- and resting tremor, and diffuse disturbances in coarse coordination of the upper extremities. Finally, the patient developed sudden onset of immobilizing vertigo.

Eight weeks after the tick bite, blood laboratory evaluation was repeated. CBC, CRP, electrolytes, liver enzymes and creatinin were all still within normal limits. But at that time the results showed reactive borreliosis (Borrelia screening test: enzyme-linked immunosorbent assay (ELISA)-Immunoglobulin G (IgG)-antibody > 200 U/ml and ELISA-IgM-antibody >100 U/ml; normally each < 9 U/ml; Borrelia confirmation test: Western Blot-IgG and -IgM were also positive, reaction against OspC in IgM-antibody-classes, and against 18kD, OspC and VlsE in the IgG-antibody-classes; Medica, Medical Laboratories Dr. F. Kaeppeli AG, Switzerland).

Lumbar puncture was subsequently performed. Cerebrospinal fluid (CSF) showed an increased protein count (2.006 g/l, normally 0.2-0.4 g/l), increased lactate level (2.5 mmol/l, normally 1.2-2.1 mmol/l), slightly lowered glucose level (2 mmol/l, normally 2.4-4.2 mmol/l) and an increased lymphocyte (252 cells/ul, 99.5% lymphocytes) and IgG-antibody count (0.353 g/l, normally <0.051 g/l). Further, CSF tests for viral agents such as early summer meningoencephalitis (ESME), human immunodeficiency virus (HIV), cytomegalovirus (CMV), enterovirus, epstein-barr-virus (EBV), herpes simplex virus (HSV) and varicella zoster virus (VZV) were negative. However, reactive *Borrelia burgdorferi* IgG- and IgM-antibodies were positive using an enzyme immunoassay (EIA) test (Immunoblot IgG with recombinant antigens: *B. burgdorferi* VIsE and p41 positive and Immunoblot IgM with recombinant antigens: *B. burgdorferi* VIsE, p39, and OspC positive; Institute for Medical microbiology, University Hospital of Zurich, Switzerland).

**Figure 1:**
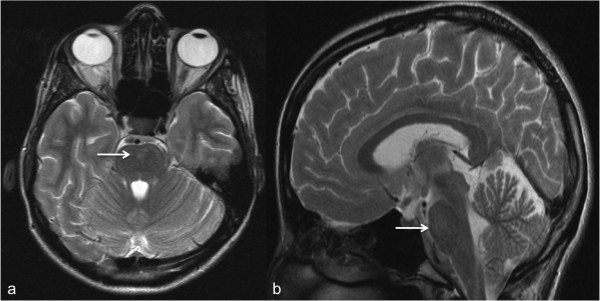
**Brainstem abnormalities in a 28-year-old patient. (a)** Axial and **(b)** sagittal T2w- TSE MR- images of the brain show hyperintense signal alterations in the pons (arrows). T2w, T2-weighted; TSE, turbo spin-echo; MR, magnetic resonance.

**Figure 2:**
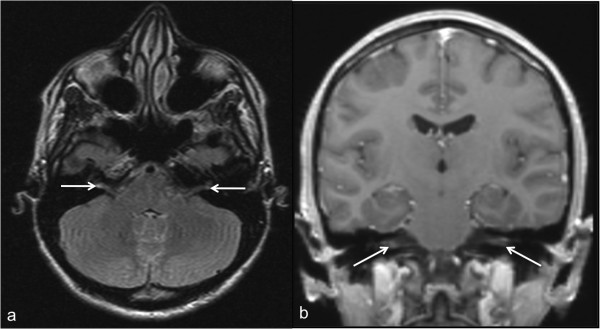
**Hyperintense signal alterations and post-contrast vestibular nerve root enhancement in a 28-year-old patient. (a)** Axial T2w dark fluid image shows bilateral hyperintense signal in the vestibular nerves. **(b)** Coronal T1w, post-contrast image shows bilateral enhancement of the vestibular nerves.

An MRI of the brain was performed using a 1.5 Tesla MRI unit (Vision, Siemens, Medical Solutions, Erlangen, Germany). Mild hyperintense lesions on T2w TSE images were visible in the pons (Figure 1). Furthermore, strong bilateral T2w hyperintense signal alterations and post-contrast enhancement of the vestibular nerves within the internal auditory canal was noted (Figure 2). No meningeal enhancement, nor any diffusion restrictions were noted.

Therapy with intravenous ceftriaxone for three weeks was initiated. All symptoms resolved.

Here, we present a patient with hyperintense lesions in the pons and vestibular nerves as well as bilateral vestibular nerves post-contrast enhancement on MRI in a patient diseased with early stage II neuroborreliosis who presented with unspecific encephalopathic symptoms, without the characteristic erythema chronicum migrans and initially normal laboratory parameters but delayed positive reactive Borrelia IgM- and IgG-antibodies.

There are only a few cases reporting neuroborreliosis with brainstem abnormalities. In a recent case report, hyperintense lesions in the pons in a patient with neuroborreliosis having a 2-month history of neck pain, wasting, and fatigue followed by gait disturbance, dysarthria and dysmetria were shown [7]. Another report has described a case of borreliosis involving the brainstem in a 28-year-old man, showing symmetric patchy areas of hyperintensity involving the cerebellar peduncles eventually extending to the pons and cerebellar white matter [8]. Unfortunately, no contrast media was administered in this case [8].

Meningeal and/or cranial nerve enhancement has been reported thus so far. Right trigeminal nerve enhancement in a 15-year-old boy was previously described in a case report [5]. Further, simultaneous enhancement and thickening of the third and sixed cranial nerve [9] in a 57-year-old woman and also bilateral enhancement of cranial nerves III-V, as well as of cranial nerves VII and VIII on the left side in a 12-year-old girl [10] have been reported.

A retrospective study of 66 patients revealed that positive neuroimaging findings on MRI of patients with neuroborreliosis are relatively unusual and the authors concluded that findings are usually focal lesions in the white matter of the brain or nerve-root or meningeal enhancement [4]. Unlike previously reported studies, nerve-root or meningeal enhancement was detected in a substantial percentage of patients [4].

## Conclusion

Patients with neuroborreliosis may present with unspecific neurologic symptoms and an MRI as a noninvasive imaging tool showing brain stem abnormalities and/or nerve root enhancement may help in establishing the diagnosis, especially since *Borrelia* antigens/antibodies are not routinely assessed in CSF. If the disease is missed and left untreated it might end in devastating consequences.

## Consent

Written informed consent was obtained from the patient for the publication of this report and any accompanying images.

## Competing interest

The authors declare that they have no competing interests.

## Authors’ contributions

All authors drafted and corrected the manuscript. All authors read and approved the final manuscript.
